# Enlarging the Arsenal of Test Species for Sediment Quality Assessment

**DOI:** 10.1007/s00128-023-03691-y

**Published:** 2023-02-15

**Authors:** N. Wieringa, S. T. J. Droge, A. M. Bakker, R. A. Melkert, B. J. Prast, P. F. M. Verdonschot, M. H. S. Kraak

**Affiliations:** 1 Department of Freshwater and Marine Ecology (FAME), Institute for Biodiversity and Ecosystem Dynamics (IBED), University of Amsterdam, Science Park 904, 1098 XH Amsterdam, The Netherlands; 2 Wageningen Environmental Research, Wageningen University and Research, P.O. Box 47, 6700 AA Wageningen, The Netherlands

**Keywords:** Whole sediment bioassay, *Sericostoma personatum*, *Chironomus riparius*, *Asellus aquaticus*

## Abstract

Since only a few standard benthic test species are available for sediment quality, our study aimed to employ multiple test species representing different sensitivity categories in the quality assessment of contaminated sediments. To this end three macroinvertebrate species, *Sericostoma personatum* (caddisfly, sensitivity category 10), *Asellus aquaticus* (isopod, category 3) and *Chironomus riparius* (chironomid, category 2), were exposed to sediments originating from various contamination sources in whole sediment bioassays using intact sediment cores. The agricultural sediment caused insect mortality, the agricultural and urban sediment caused isopod growth reduction and the urban and Wastewater Treatment Plant (WWTP) sediment affected chironomid emergence time. It is concluded that the arsenal of standard species can be successfully expanded by non-standard species, reducing over- or underestimation of the risks of contaminated sediments.

**Supplementary Information:**

The online version contains supplementary material available at 10.1007/s00128-023-03691-y.

## Introduction

Contaminated sediments are ubiquitous and are considered to be the largest chemical repositories on earth (Burton 2013). Hence, contaminated sediments present serious ecotoxicological risks to benthic communities, affecting both aquatic ecosystem structure and functioning (de Castro-Català et al. 2016; van der Lee et al. 2020; Wieringa et al. 2022), emphasizing the need to incorporate sediment quality into the assessment of aquatic ecosystem health.

In traditional sediment quality assessments, like the TRIAD approach, chemical analysis, in situ community composition and laboratory whole sediment bioassays are combined (Chapman et al. 1997). For the laboratory bioassays, however, generally only one test species is selected, for example a chironomid or a worm. Yet, different species respond differently to the same contaminated sediment (Brock et al. 2020). These species-specific sensitivities to contaminated sediments may be caused by differences between benthic invertebrates in feeding habit, life stage, size, and uptake route of sediment associated compounds (Tuikka et al. 2011). Hence, employing only a single test species may either overestimate or underestimate the risks of contaminated sediments for benthic communities. It is therefore recommended to compose bioassay batteries with a variety of test species, assessing the effects on various endpoints in order to represent natural communities as much as possible (Brock et al. 2020; de Baat et al. 2019; Tuikka et al. 2011).

A major drawback of the few standard test organisms currently available for freshwater sediment quality assessment, such as the chironomids *Chironomus riparius* and *Chironomus tentans* (OECD 2004a) and worms (OECD 2008), is that these species generally receive a low quality score in classical water quality assessment systems (Hawkes 1998). Other tests species, like nematodes, are not part of common water quality assessment systems in Europe, or originate from other continents, like the amphipod *Hyalella azteca* and thus do not represent local communities. Therefore, the set of species employed in whole sediment bioassays should be expanded by other (non)standard species, representing the more sensitive categories of traditional assessment approaches.

The aim of the present study was therefore to employ multiple test species representing different sensitivity categories in the quality assessment of contaminated sediments. To this end three macroinvertebrate species, the caddisfly *Sericostoma personatum*, the isopod *Asellus aquaticus* and the chironomid *Chironomus riparius*, were exposed to sediments originating from various contamination sources in whole sediment bioassays using intact sediment cores. Hence, in the present study one standard test species and two non-standard test species were explored. As standard sediment test organism the non-biting midge *Chironomus riparius* was selected, allowing to monitor multiple (sub)lethal endpoints (OECD 2004a), that are indicative of relevant sediment contamination levels (de Baat et al. 2019). The chironomids represent the category of relatively insensitive species, having a score of 2 out of 10 in the Biological Monitoring Working Party (BWMP) index (Hawkes 1998). The isopod *Asellus aquaticus* was the second candidate, since it has already been frequently employed as freshwater test organism, but it is not yet a standard test organism in ecotoxicity tests (O’Callaghan et al. 2019). Asellidae have a score of 3 out of 10 in the BWMP index (Hawkes 1998). As potential new test organisms the sediment inhabiting larvae of the caddisfly *Sericostoma personatum* were selected. Caddisflies (Trichoptera) are frequently used to assess ecological and ecotoxicological water quality (e.g. Resh 1992), but not for sediment quality assessment. Species belonging to the caddisfly family Sericostomatidae have a score of 10 out of 10 in the BWMP index (Hawkes 1998). Based on the BWMP scores of the three selected species, it was hypothesized that they would respond differently to contaminated sediments.

## Materials and methods

Sediment sampling locations were selected based on prior research in collaboration with the Dutch water authorities (de Baat et al. 2019). In that study, the predominant surrounding land use and pollution source comprised three categories: urban, Wastewater Treatment Plant (WWTP) effluent, and agriculture. Sampling site information can be found in Table S1, physical characterization in Table S2. The total concentrations of the selected metals were determined in the sediment extracts, after which freely dissolved pore water concentrations were calculated. Passive sampling with solid-phase micro-extraction (SPME) fibres was applied to determine the pore water concentrations of selected organic compounds. The analytical methods and the respective limits of detection and limits of quantification can be found in Table S3 and S4 and the chemical profiling of the sediments in Table S5. Toxic units (TU) were calculated as toxicity index (SI1). In the present study each pollution source was represented by one specific location in addition to a reference site. The reference site was situated on the University of Amsterdam Science Park campus with no apparent pollution source (TU = 0.05). The agricultural site was located between greenhouses, where a wide array of pesticides has been used (TU = 0.14). The WWTP site received effluent from the treatment plant of the city of Hilversum (capacity of 120.000 inhabitant equivalents per day) causing a high and continuous loading of contaminants to the receiving surface water and sediment (TU = 0.65). The urban site was located in the city of Amsterdam and contained high levels of polycyclic aromatic hydrocarbons (PAHs) and metals (TU = 0.7). Per location 20 intact sediment cores were sampled in April 2018. Each location was sampled on a single occasion using a sediment core sampler (UWITEC, Mondsee, Austria) loaded with acrylic tubes (l: 60 cm, d: 6 cm) if the water level did not allow to collect samples by hand. Sediment cores were prepared in the laboratory according to the method described in de Baat et al. (2019). Sediment cores were stored at − 20 °C for at least 48 h before the start of the experiments to eliminate indigenous fauna. Information on collection of test organisms and culture set up can be found in SI2 and Table S6.

In addition to the reference site a negative laboratory control was included in the experimental setup as well, consisting of artificial sediment according to OECD guideline 218 (OECD 2004a) with slight modifications (Marinković et al. 2011) containing 140 mg food (a mixture of Trouvit and Tetraphyll in a ratio of 20:1) per core, representing 1 mg/larva/day food for the entire duration of the experiment. The artificial sediment was sterilized by autoclaving and homogenized in glass bottles on a roller bank at 20 rpm for > 24 h.

One day prior to the experiment, the cores were thawed, topped off with Dutch standard water and aeration was turned on to saturate the water overlying the sediment cores with oxygen. The *S. personatum* 28 d whole sediment bioassays were performed with field collected individuals available in sizes ranging from 0.3 to 2.0 cm. There were four replicates per treatment. At the start of the experiment, five *S. personatum* larvae were added per replicate sediment core. The larvae were fed according to dos Reis Oliveira et al. (2018) and Hutchens et al. (1997), with adaptation of the amount of food at the start of the experiment, when 0.98 ± 0.16 g (n = 20) incubated oak and birch leaves and 70 mg of a solution of a mixture of Trouvit and TetraPhyll (20:1) were added. After 14 days, 35 mg of the food solution, but no leaves, was added. Demineralized water was regularly added to all cores to compensate for water loss by evaporation. At the end of the 28 d experiment, the number of surviving larvae was counted.

The *Asellus aquaticus* 28 d whole sediment bioassays were performed with field collected juvenile isopods (< 2.5 mm). There were five replicates per treatment. The set-up, feeding and negative laboratory control was the same as for *S. personatum* with adaptation of the amount of food at the start of the experiment. Five juveniles per replicate sediment core and 0.57 ± 0.2 g (n = 25) oak leaves incubated for seven days in stream water were added at the start of the experiment, and after 14 days, 0.2 ± 0.05 g (n = 25) incubated oak leaves were added. Demineralized water was regularly added to the cores to compensate for water loss by evaporation. At the end of the 28 d experiment, the number of surviving isopods was counted and their length was measured using a stereo microscope (Leica M165C, Wetzlar, Germany). Individual isopod growth was calculated by subtracting the initial average length, obtained by measuring the length of 10 randomly selected individuals prior to the experiment, from the individual final length.

The *C. riparius* 28 d whole sediment bioassays were performed with first instar larvae (< 24 h) from an in-house culture, based on OECD guideline 218 (OECD 2004a; de Baat et al. 2019). There were ten replicates per treatment. In addition to the reference site a negative laboratory control (n = 5) was included in the experimental setup as well. Set-up and negative laboratory control was the same as for *S. personatum*, but because of the double number of larvae, the food per core added for the entire duration of the experiment was amounted to 0.5 mg/larva/day food (a solution of a mixture of Trouvit and TetraPhyll (20:1)). At the start of the experiment ten larvae per replicate sediment core were added. After 7 and 14 days, 17.5 mg of additional food was added, corresponding with 0.25 mg food/larva/day for a period of 7 days (OECD 2004a). After 14 days, the sediment cores were covered with fine mesh gauze and checked daily for emerging midges, which were sexed and removed. If needed, demineralized water was added daily to compensate for evaporation losses. At the end of the 28 d experiment, the sediments were sieved (350 μm) and the surviving larvae were counted. Survival (the number of emerged adults and surviving larvae), the number of emerged adults and the emergence time of the adults were assessed as endpoints (OECD 2004a).

In all bioassays, dissolved oxygen concentration, conductivity, and pH were measured in the overlying water of each core at the start of the experiment and after 14 and 28 days using a benchtop multimeter (HACH, Tiel, The Netherlands). The ammonium concentration was also determined at the start of the experiment and after 14 and 28 days by analyzing 1 mL of filtered (0.2 μm pore size) overlying water of each core on an Autoanalyzer (SAN++, Skalar, Breda, The Netherlands).

The artificial sediment served as a negative control to check the test performance and to test the organisms’ viability. Performance of the three benthic invertebrate test species on the contaminated sediments was therefore compared to that on the reference sediment. A General Linear Model (GLM) was applied to test for statistical differences between the reference and contaminated sediments for all species and endpoints using R-studio (version 3.4.3). As a binomial distribution was assumed for survival and emergence data, we used the binomial family (link function =’logit’). For growth data of *Asellus aquaticus*, a nested GLM was used, as it includes individuals within replicates and the data were Gaussian distributed. For *C. riparius*, the EmT50, i.e. the day at which 50% emergence occurred, was calculated for each treatment by plotting the cumulative number of emerged midges against time. Since emergence times differ between males and females, this was done separately for each gender. A non-linear regression was conducted using the logistic curve with the EmT50 as parameter. Significant differences between the reference site and all other treatments were checked using a one-way ANOVA with Dunnett’s multiple comparison post-hoc test with significance level at p < 0.05. Statistical analyses were performed in GraphPad Prism®, Version 5.00 for EmT50 calculations.

## Results

The pH, temperature and ammonium concentration in the whole sediment bioassays in all cores and the emergence of the chironomids in the control and the reference cores were in accordance with the validity criteria of OECD guideline 218 (OECD 2004a) (Table S7). Four cores (1x laboratory control, 1x reference, 2x WWTP) from the *C. riparius* experiment were excluded from further analyses due to a surplus of one extra test organism.

**Fig. 1:**
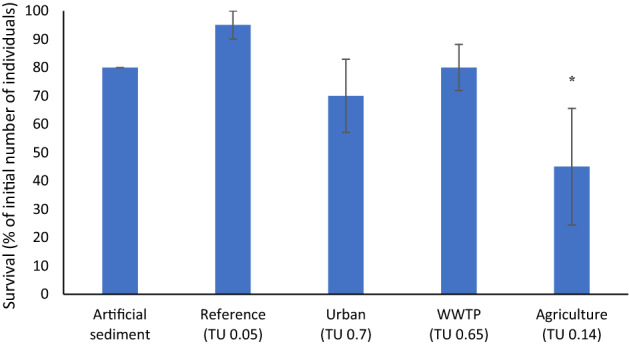
Survival (mean ± SE; % of initial number of individuals) of *S. personatum* after 28 d of exposure to artificial sediment and intact sediment cores from the reference, urban, WWTP and agricultural site (n = 4 per treatment). An asterisk indicates a significant (*p* < 0.05) difference

Control survival of *S. personatum* was 80%. Caddisfly survival on sediment from the agricultural site (45%) was significantly (*p* < 0.01) lower than on the reference sediment (95%) (Fig. 1).

**Fig. 2:**
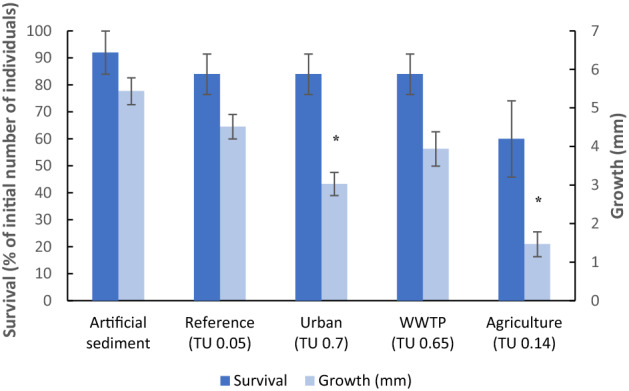
Survival (dark blue bars, mean ± SE; % of initial number of individuals) and growth (light blue bars, mean ± SE; in mm) of *Asellus aquaticus* after 28 d of exposure to the artificial sediment and intact sediment cores from reference, urban, WWTP and agricultural site (n = 5 per treatment). An asterisk indicates a significant (*p* < 0.05) difference

Control survival of *Asellus aquaticus* in the 28 d whole sediment bioassays on artificial sediment was 92% and control growth 5.4 mm ± 0.3 mm (mean ± SE). Isopod survival was not significantly (*p* > 0.05) impacted by the urban sediment (84%), WWTP sediment (84%) and sediment from the agricultural site (60%) compared to the reference sediment (84%) (Fig. 2). In contrast to survival, growth of *Asellus aquaticus* was significantly (*p* < 0.05) lower at the urban sediment (3.0 mm, ± 0.3 mm, *p* < 0.01) and the agricultural sediment (1.5 mm, ± 0.3 mm, *p*  < 0.001) compared to the reference sediment (4.5 mm ± 0.3 mm) (Fig. 2). Hence, two of the three contaminated sediments affected isopod growth.

**Fig. 3:**
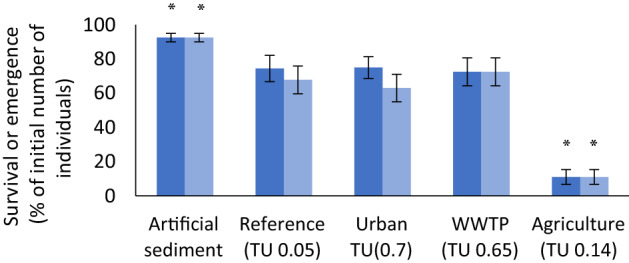
Survival (dark blue bars) and emergence (light blue bars) (mean ± SE; % of initial number of individuals) of *C. riparius* after 28 d of exposure to intact sediment cores from the artificial sediment, reference, urban, WWTP and agricultural site (n = 10 per treatment). An asterisk indicates a significant (*p* < 0.05) difference

Control survival and emergence of *C. riparius* in the 28 d whole sediment bioassays on artificial sediment was both 93% ± 2.5 (mean ± SE). Survival (11% ± 4.3, *p* < 0.0001) and emergence (11% ± 4.3, *p* < 0.0001) on the agricultural sediment were significantly lower than on the reference sediment (survival 74% ± 7.7; emergence 68% ± 8.1) (Fig. 3).

**Fig. 4:**
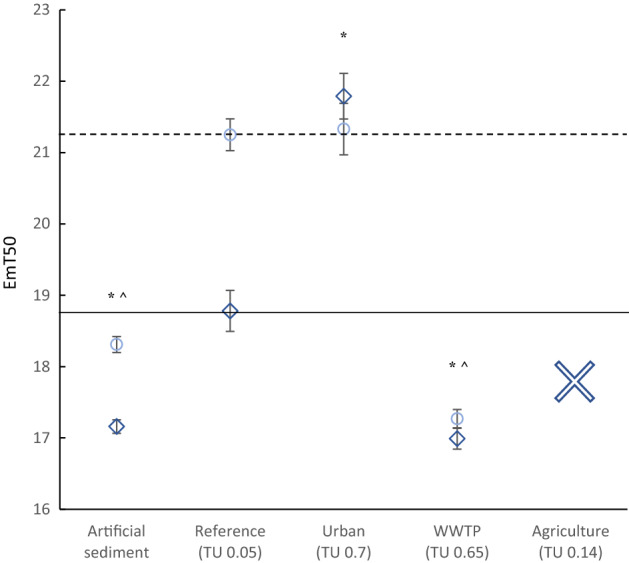
Median (± SE) 50% emergence time in days (EmT50) of *C. riparius* males (dark blue triangles) and females (light blue circles) after 28 d of exposure to intact sediment cores from the artificial sediment, reference, urban, WWTP and agricultural site (cross = not applicable), (n = 10 per treatment). The horizontal lines represent the EmT50 value for the reference sediment (straight: males, dotted: females), to which the other locations were compared. An asterisk (males) or caret (females) indicates a significant (*p* < 0.05) difference

Generally, male and female chironomids show a bimodal emergence pattern with females emerging later than males (Fig. 4). The control EmT50 of *C. riparius* males and females in the 28 d whole sediment bioassays was 17.6 days ± 0.1 (mean ± SE, *p* < 0.001) and 18.3 days ± 0.1, respectively (*p* < 0.001). The EmT50 value for male and female midges on the reference sediment was 18.8 days ± 0.3 and 21.3 days ± 0.2, respectively. For males, the EmT50 value at the urban site (21.8 days ± 0.3, *p* < 0.001) was longer than at the reference site, indicating delayed midge emergence, while for female midges, this was not observed (21.3 days ± 0.4 (*p* > 0.05). In contrast, the EmT50 values for both males and females on the WWTP sediment (resp. 17.0 days ± 0.1,  *p* < 0.001 and 17.3 days ± 0.1 (*p* < 0.001) were shorter than on the reference sediment, indicating accelerated emergence.

## Discussion

Since the number of standardized test species for sediment toxicity testing with benthic invertebrates is rather limited, in the present study two non-standardized test species were employed. The first attempt to use *S. personatum* as test organism in whole sediment bioassays was rather successful, as field collection was fairly easy, control survival on artificial sediment and on reference sediment was high, while the agricultural sediment caused severe mortality of the caddisfly larvae. Moreover, a laboratory culture of *S. personatum* could be set up to ensure a constant availability of larvae in similar life stages. This paves the way for this species towards incorporation into sediment quality assessment, preferably supported by the development of a standard test protocol with sublethal endpoints in addition to survival. The isopod *Asellus aquaticus* has been used in field and laboratory sediment toxicity studies before (Brock et al. 2020; dos Reis Oliveira et al. 2018), but it is not a standard test organism yet. Field collection was fairly easy, control survival on artificial sediment and on reference sediment was high, while on the contaminated sediments growth was significantly affected. Also for isopods laboratory cultures could be set up. The successful use of *Asellus aquaticus* in the present study and by other researchers show that this organism is qualified to be deployed as a standard test species for whole sediment bioassays. Among the three selected test species, *C. riparius* was the only standardized test species for sediment toxicity testing (OECD 2004a, b) and proved to be a useful test organism for whole sediment bioassays showing lethal as well as subtle sublethal effects. The use of *C. riparius* allowed for the monitoring of multiple (sub)lethal endpoints (de Baat et al. 2019). The emergence pattern observed in our study corresponded to the results of previous research (de Baat et al. 2019).

Our results showed that each test species brings its own complementary information. *C. riparius* and *S. personatum* showed decreased survival on the agricultural sediment, while *Asellus aquaticus* did not. However, isopod growth did respond to the agricultural sediment as well as to the urban sediment. *C. riparius* showed effects on the sublethal endpoint EmT50, with differences in emergence time at the urban (males-delayed) and WWTP (both males and females-accelerated) sediment, thus providing additional information. Hence, the three test species responded differently to the contaminated sediments and therefore provided additional and complementary insights, especially considering the various sublethal endpoints. It is therefore concluded that the caddisfly *S. personatum* and the isopod *Asellus aquaticus* would be welcome sediment test species in addition to the traditional test species such as the non-biting midge *C. riparius* and the worm *L. variegatus*.

In the present study, multiple test species were used, representing different sensitivity categories in traditional water quality assessment, respectively 2 (Chironomidae), 3 (Asellidae) and 10 (Sericostomatidae) (Hawkes 1998). Toxic units (TU) were calculated as toxicity index, to substantiate the term land use. However, it must be emphasised the TUs only takes the measured compounds into account. At the WWTP site (TU = 0.65), the observed responses to contaminated sediment matched with this water quality-based classification. Here, there was no effect on survival of any test species, but isopod growth was adversely affected, while chironomid emergence time was accelerated. Also at the urban site (TU = 0.7) no mortality was observed and there was also no effect on the number of emerged chironomids. Only if the more sensitive sublethal endpoint emergence time was considered adverse effects were noticed, while isopod growth was again also affected. When the present contaminants had a specific mode of action, like in the agricultural sediment (TU = 0.14), all test organisms were affected. The two insect species were, however, more severely affected than the isopod, since the insects hardly survived, while the isopod experienced only sublethal effects. Hence, the presence of compounds with specific modes of action, in this case most likely insecticides (Table S2), overrules the traditional sensitivity categories by affecting specific non target species (Schäfer 2019).

In the present study it was demonstrated that the two newly added species, *Asellus aquaticus* and *S. personatum*, are an informative addition to the limited number of benthic test organisms and that they do well in whole sediment bioassays. Expanding the range of test species would further strengthen bioassay-based toxicity assessment of contaminated sediments. This may be achieved by selecting more species with different sensitivities according to e.g. the BWMP index (Hawkes 1998) or by selecting species that complement each other in terms of ecological traits. Although a bioassay battery is preferred to reduce over- and under estimation of the risks of contaminated sediments, time and financial constraints may force water authorities to make a customised selection of the available test species, facilitated by the present successful addition of non-standard test species.

As hypothesized, species specific responses were observed after exposure to the various sediments, albeit not fully related to traditional sensitivity categories. Nonetheless, it is concluded that the arsenal of standard species can be successfully expanded by non-standard species.

## Supplementary Information

Below is the link to the electronic supplementary material.

Supplementary file1 (DOCX 85 KB)
