# Supplementary material for: Enlarging the Arsenal of Test Species for Sediment Quality Assessment
Source: Bull Environ Contam Toxicol. 2023 Feb 15;110(2):55. doi: 10.1007/s00128-023-03691-y (PMC9931774; doi:10.1007/s00128-023-03691-y)
Supplement: Supplementary file 1 — Supplementary file1 (DOCX 85 KB) [file 128_2023_3691_MOESM1_ESM.docx]

Supplementary Material

**Enlarging the arsenal of test species for sediment quality assessment**

Wieringa, N.^1*^, Droge, S.T.J.^1,2^, Bakker, A.M. ^1,2^, Melkert, R.A. ^1^, Prast, B.J. ^1^, Verdonschot, P.F.M. ^1,2^ & Kraak, M.H.S. ^1^

1. Department of Freshwater and Marine Ecology (FAME), Institute for Biodiversity and Ecosystem Dynamics (IBED), University of Amsterdam, Science Park 904, 1098 XH Amsterdam, The Netherlands
2. Wageningen Environmental Research, Wageningen University and Research, P.O. Box 47, 6700 AA Wageningen, The Netherlands

* Corresponding author: [n.wieringa@uva.nl](mailto:n.wieringa@uva.nl)

12 Pages

7 Tables

**Table S1.** Overview of the investigated study sites.

| **land use** | **Location** | **GPS coordinates** | **sampling day (dd/mm/yy)** |
| --- | --- | --- | --- |
| *Reference* | Science Park | 52.356795, 4.955046 | 11/04/18 |
| *Urban* | Wittenkade | 52.380458, 4.873499 | 23/04/18 |
| *WWTP* | Hilversum | 52.252807, 5.243704 | 13/04/18 |
| *Agriculture* | Westland | 51.961299, 4.192689 | 06/04/18 |

**Table S2.** Particle size distribution

All sediments were subjected to the dry sieving method (Cammeraat & Imeson, 1998). One sediment sample (>20 gr) per location was oven-dried at 30 ̊C and then fractionated using mesh sieves to obtain eight size fractions. Sieves were shaken for 5 min at 40 Hz using a horizontal shaking machine (AS 200 basic; Retsch, Aartselaar, Belgium) and each fraction was weighed. Data obtained from de Baat et al., 2019.

| **Location** | **Fractions** | | | | | | | | |
| --- | --- | --- | --- | --- | --- | --- | --- | --- | --- |
|  | **> 4,0 mm** | **2 mm** | **1 mm** | **0,5 mm** | **0,25 mm** | **>250mm** | **0,125 mm** | **0,063 mm** | **< 0,063 mm** |
| Artificial sediment | 0.0 | 0.0 | 0.0 | 2.5 | 7.9 | 10.4 | 69.0 | 7.3 | 13.3 |
| Reference | 0.4 | 2.2 | 4.4 | 16.9 | 46.1 | 69.9 | 26.0 | 2.9 | 1.1 |
| Urban | 2.0 | 1.2 | 2.7 | 7.3 | 40.5 | 53.7 | 40.8 | 4.4 | 1.1 |
| WWTP | 1.2 | 4.3 | 6.4 | 7.9 | 17.3 | 37.0 | 54.0 | 8.6 | 0.4 |
| Agriculture | 0.0 | 0.5 | 5.6 | 5.0 | 17.1 | 28.2 | 61.2 | 9.3 | 1.2 |

Cammeraat, L. H.; Imeson, A. C. Deriving indicators of soil degradation from soil aggregation studies in south eastern Spain and southern France. Geomorphology 1998, 23 (2–4), 307–321

**Table S3.** Compounds targeted in chemical profiling of the investigated sediments and their respective limits of quantification (LOQ). In fiber and freely dissolved LOQs are calculated based on logK_ow_, logK_fw_ and analytical LOQ. See for more information de Baat et al., 2019.

| **compound group** |  |  |  |  |  |  |  |  |  |  |
| --- | --- | --- | --- | --- | --- | --- | --- | --- | --- | --- |
| **inorganic** | **target compound** | **pollutant type** | **Remark** | **detector** | **LOQ** | **unit** |  |  |  |  |
| *metals* | Al | Legacy |  | ICP-OES | 24 | µg/L |  |  |  |  |
|  | As | Legacy |  | ICP-OES | 37 | µg/L |  |  |  |  |
|  | Ag | Legacy |  | ICP-OES | 1 | µg/L |  |  |  |  |
|  | Cd | Legacy |  | ICP-OES | 1 | µg/L |  |  |  |  |
|  | Cr | Legacy |  | ICP-OES | 2 | µg/L |  |  |  |  |
|  | Cu | Legacy |  | ICP-OES | 1 | µg/L |  |  |  |  |
|  | Fe | Legacy |  | ICP-OES | 1 | µg/L |  |  |  |  |
|  | Mn | legacy |  | ICP-OES | 3 | µg/L |  |  |  |  |
|  | Ni | legacy |  | ICP-OES | 4 | µg/L |  |  |  |  |
|  | Pb | legacy |  | ICP-OES | 15 | µg/L |  |  |  |  |
|  | Se | legacy |  | ICP-OES | 16 | µg/L |  |  |  |  |
|  | Zn | legacy |  | ICP-OES | 4 | µg/L |  |  |  |  |
| **compound group** |  |  |  |  |  |  | **LOQ** | | |  |
| **organic** | **target compound** | **pollutant type** | **Remark** | **detector** | **logK_ow_** | **logK_fw_** | **analytical** | **in fiber** | **freely dissolved** | **unit** |
| *PAHs* | phenanthrene | legacy |  | LC-FLU | 4.46 | 4.29 | 12 | 3908.1 | 0.200 | µg/L |
|  | pyrene | legacy |  | LC-FLU | 4.88 | 4.99 | 2 | 651.4 | 0.007 | µg/L |
| *WWTP markers* | HHCB | musk fragrance |  | GC-MS | 5.9 | 6.19 | 100 | 24450.6 | 0.016 | µg/L |
|  | bisphenol A | plastic precursor |  | LC-MS | 3.3 | 3.30 | 2 | 651.4 | 0.326 | µg/L |
|  | nonylphenol | surfactant precursor | isomer mixture | GC-MS | 5.15 | 5.36 | 100 | 24450.6 | 0.108 | µg/L |
|  | triclosan | antimicrobial agent |  | GC-MS | 4.3 | 4.41 | 100 | 24450.6 | 0.949 | µg/L |
|  | mehtyl-triclosan | metabolite |  | GC-MS | 5 | 5.19 | 100 | 24450.6 | 0.158 | µg/L |
| *pesticides* | 2,4,5-trichlorophenoxyacetic acid | herbicides | dissociated | LC-MS | 4 | 4.08 | 6 | 1954.1 | 0.163 | µg/L |
|  | 2,4-dichlorophenoxyacetic acid | herbicides | dissociated | LC-MS | 2.81 | 2.76 | 4 | 1302.7 | 2.287 | µg/L |
|  | dichlorprop | herbicides | dissociated | LC-MS | 3.43 | 3.44 | 4 | 1302.7 | 0.468 | µg/L |
|  | 2,6-di-tert-butyl-4-methylphenol | n.a. |  | GC-MS | 5.1 | 5.30 | 250 | 81418.8 | 0.408 | µg/L |
|  | 4-(2,4-dichlorophenoxy)butyric acid | herbicides | dissociated | LC-MS | 3.53 | 3.56 | 5 | 1628.4 | 0.453 | µg/L |
|  | 4-chloroaniline | n.a. |  | GC-MS | 1.83 | 1.67 | 50 | 16283.8 | 350.823 | µg/L |
|  | 4-chlorophenoxyacetic acid | herbicides | dissociated | LC-MS | 2.25 | 2.13 | 4 | 1302.7 | 9.583 | µg/L |
|  | 4-nonylphenol | n.a. |  | GC-MS | 5.76 | 6.03 | 10 | 3256.8 | 0.003 | µg/L |
|  | 4-tert-octylphenol | n.a. |  | GC-MS | 5.25 | 5.47 | 25 | 8141.9 | 0.028 | µg/L |
|  | acetamiprid | insecticides |  | LC-MS | 0.8 | 0.52 | 0.3 | 97.7 | 29.355 | µg/L |
|  | aclonifen | herbicides |  | GC-MS | 4.04 | 4.12 | 500 | 162837.7 | 12.289 | ng/L |
|  | alachlor | herbicides |  | GC-MS | 3.52 | 3.54 | 150 | 48851.3 | 13.945 | ng/L |
|  | atrazine | herbicides |  | GC-MS | 2.61 | 2.53 | 100 | 32567.5 | 95.379 | ng/L |
|  | azinphos-ethyl | insecticides |  | GC-MS | 3.18 | 3.17 | 250 | 81418.8 | 55.470 | ng/L |
|  | azinphos-methyl | insecticides |  | GC-MS | 2.75 | 2.69 | 750 | 244256.5 | 499.985 | ng/L |
|  | azoxystrobin | fungicides |  | LC-MS | 2.5 | 2.41 | 0.9 | 293.1 | 1.137 | µg/L |
|  | bentazon | herbicides |  | LC-MS | 2.8 | 2.74 | 3 | 977.0 | 1.760 | µg/L |
|  | bifenox | herbicides |  | GC-MS | 4.48 | 4.61 | 1.25 | 407.1 | 0.010 | µg/L |
|  | bixafen | fungicides |  | LC-MS | 3.3 | 3.30 | 0.8 | 260.5 | 0.131 | µg/L |
|  | boscalid | fungicides |  | LC-MS | 2.96 | 2.92 | 2 | 651.4 | 0.779 | µg/L |
|  | carbendazim | fungicides |  | LC-MS | 1.48 | 1.28 | 2 | 651.4 | 34.359 | µg/L |
|  | carfentrazone-ethyl | herbicides |  | GC-MS | n.a. | n.a. | 150 | 48851.3 | n.a. | ng/L |
|  | chlorbromuron | herbicides |  | LC-MS | 3.09 | 3.07 | 5 | 1628.4 | 1.397 | µg/L |
|  | chlorfenvinphos | insecticides |  | GC-MS | 3.81 | 3.87 | 500 | 162837.7 | 22.135 | ng/L |
|  | chloridazon | herbicides |  | LC-MS | 1.14 | 0.90 | 0.5 | 162.8 | 20.500 | µg/L |
|  | chlorothalonil | fungicides |  | GC-MS | 2.94 | 2.90 | 125 | 40709.4 | 51.250 | µg/L |
|  | chlorotoluron | herbicides |  | LC-MS | 2.41 | 2.31 | 3 | 977.0 | 4.773 | µg/L |
|  | chlorpropham | herbicides |  | LC-MS | 2.96 | 2.92 | 2 | 651.4 | 0.779 | µg/L |
|  | chlorpyrifos | insecticides |  | GC-MS | 4.96 | 5.14 | 750 | 244256.5 | 1.751 | ng/L |
|  | cis-1,2,3,6-tetrahydrophthalimide | n.a. |  | GC-MS | 0.3 | -0.03 | 12.5 | 4070.9 | 4395.702 | µg/L |
|  | clomazone | herbicides |  | LC-MS | 2.5 | 2.41 | 0.6 | 195.4 | 0.758 | µg/L |
|  | clothianidin | insecticides |  | LC-MS | 0.905 | 0.64 | 10 | 3256.8 | 747.990 | µg/L |
|  | coumaphos | insecticides |  | LC-MS | 4.13 | 4.22 | 0.4 | 130.3 | 0.008 | µg/L |
|  | cyazofamid | fungicides |  | LC-MS | 3.2 | 3.19 | 4 | 1302.7 | 0.843 | µg/L |
|  | cycloxydim | herbicides |  | LC-MS | 3.31 | 3.31 | 0.3 | 97.7 | 0.048 | µg/L |
|  | cymoxanil | fungicides |  | LC-MS | 0.67 | 0.38 | 3 | 977.0 | 409.382 | µg/L |
|  | cypermethrin | insecticides |  | GC-MS | 6.6 | 6.97 | 225 | 73276.9 | 0.008 | ng/L |
|  | cyproconazole | fungicides |  | LC-MS | 3.09 | 3.07 | 3 | 977.0 | 0.838 | µg/L |
|  | DEHP | n.a. |  | GC-MS | 7.6 | 8.08 | 250 | 81418.8 | 0.001 | µg/L |
|  | deltamethrin | insecticides |  | GC-MS | 6.2 | 6.52 | 250 | 81418.8 | 0.024 | ng/L |
|  | desethylterbuthylazine | herbicides |  | GC-MS | 2.23 | 2.11 | 500 | 162837.7 | 1260.793 | ng/L |
|  | desmedipham | herbicides |  | LC-MS | 3.39 | 3.40 | 2 | 651.4 | 0.259 | µg/L |
|  | diazinon | insecticides |  | GC-MS | 3.81 | 3.87 | 50 | 16283.8 | 2.214 | ng/L |
|  | dichlofluanid | fungicides |  | GC-MS | 3.7 | 3.74 | 200 | 65135.1 | 11.732 | ng/L |
|  | dichlorvos | insecticides |  | GC-MS | 1.43 | 1.22 | 50 | 16283.8 | 976.186 | ng/L |
|  | dicofol | insecticides |  | GC-MS | 4.3 | 4.41 | 50 | 16283.8 | 0.632 | ng/L |
|  | DEET | insect repellant |  | GC-MS | 2.18 | 2.06 | 500 | 162837.7 | 1432.845 | ng/L |
|  | difenoconazole | fungicides |  | GC-MS | 4.36 | 4.48 | 1.25 | 407.1 | 0.014 | µg/L |
|  | diflufenican | herbicides |  | GC-MS | 4.9 | 5.08 | 500 | 162837.7 | 1.361 | ng/L |
|  | dimethanamid | herbicides |  | LC-MS | 1.89 | 1.73 | 0.7 | 228.0 | 4.213 | µg/L |
|  | dimethoate | insecticides |  | GC-MS | 0.78 | 0.50 | 125 | 40709.4 | 12873.448 | ng/L |
|  | dimethomorph | fungicides |  | GC-MS | 2.68 | 2.61 | 150 | 48851.3 | 119.609 | ng/L |
|  | disulfoton | insecticides |  | GC-MS | 4.02 | 4.10 | 1 | 325.7 | 0.026 | µg/L |
|  | diuron | herbicides |  | LC-MS | 2.68 | 2.61 | 4 | 1302.7 | 3.190 | µg/L |
|  | epoxiconazole | fungicides |  | LC-MS | 3.3 | 3.30 | 1 | 325.7 | 0.163 | µg/L |
|  | esfenvalerate | insecticides |  | GC-MS | 6.22 | 6.54 | 500 | 162837.7 | 0.046 | ng/L |
|  | ethofumesate | herbicides |  | LC-MS | 2.7 | 2.63 | 10 | 3256.8 | 7.576 | µg/L |
|  | ethoprophos | insecticides |  | GC-MS | 3.59 | 3.62 | 50 | 16283.8 | 3.886 | ng/L |
|  | fenamiphos | insecticides |  | GC-MS | 3.23 | 3.22 | 750 | 244256.5 | 146.428 | ng/L |
|  | fenitrothion | insecticides |  | GC-MS | 3.3 | 3.30 | 75 | 24425.6 | 12.242 | ng/L |
|  | fenoxycarb | insecticides |  | GC-MS | 4.3 | 4.41 | 1.25 | 407.1 | 0.016 | µg/L |
|  | fenpropidin | fungicides | protonated | LC-MS | 2.6 | 2.52 | 2 | 651.4 | 1.957 | µg/L |
|  | fenpropimorph | fungicides |  | GC-MS | 4.5 | 4.63 | 100 | 32567.5 | 0.758 | ng/L |
|  | fenthion | insecticides |  | GC-MS | 4.091 | 4.18 | 75 | 24425.6 | 1.618 | ng/L |
|  | fipronil | insecticides |  | LC-MS | 4 | 4.08 | 3 | 977.0 | 0.082 | µg/L |
|  | flonicamid | insecticides |  | LC-MS | -0.24 | -0.63 | 9 | 2931.1 | 12599.714 | µg/L |
|  | florasulam | herbicides |  | LC-MS | -1.22 | -1.72 | 0.3 | 97.7 | 5153.809 | µg/L |
|  | fluazifop-p-butyl | herbicides |  | GC-MS | 4.5 | 4.63 | 125 | 40709.4 | 0.947 | ng/L |
|  | fluazinam | fungicides |  | LC-MS | 4.03 | 4.11 | 2 | 651.4 | 0.050 | µg/L |
|  | fluopicolide | fungicides |  | LC-MS | 2.9 | 2.86 | 0.4 | 130.3 | 0.182 | µg/L |
|  | fluoxastrobin | fungicides |  | LC-MS | 2.86 | 2.81 | 0.4 | 130.3 | 0.201 | µg/L |
|  | fluroxypyr | herbicides | dissociated | LC-MS | 2.2 | 2.08 | 5 | 1628.4 | 13.614 | µg/L |
|  | flutolanil | fungicides |  | LC-MS | 3.17 | 3.16 | 0.4 | 130.3 | 0.091 | µg/L |
|  | heptenophos | insecticides |  | GC-MS | 2.32 | 2.21 | 50 | 16283.8 | 100.148 | ng/L |
|  | imazalil | fungicides |  | LC-MS | 2.56 | 2.48 | 4 | 1302.7 | 4.336 | µg/L |
|  | imidacloprid | insecticides |  | LC-MS | 0.57 | 0.27 | 0.6 | 195.4 | 105.747 | µg/L |
|  | iodosulfuron-methyl-sodium | herbicides | dissociated | LC-MS | -0.7 | -1.14 | 0.5 | 162.8 | 2270.907 | µg/L |
|  | ioxynil | herbicides | dissociated | LC-MS | 0.9 | 0.63 | 3 | 977.0 | 227.286 | µg/L |
|  | iprodione | fungicides |  | GC-MS | 3 | 2.97 | 500 | 162837.7 | 175.828 | ng/L |
|  | irgarol | herbicides |  | GC-MS | 4.07 | 4.16 | 500 | 162837.7 | 11.381 | ng/L |
|  | isoproturon | herbicides |  | LC-MS | 2.84 | 2.79 | 3 | 977.0 | 1.589 | µg/L |
|  | kresoxim-methyl | fungicides |  | GC-MS | 3.4 | 3.41 | 75 | 24425.6 | 9.478 | ng/L |
|  | lambda-cyhalothrin | insecticides |  | GC-MS | 6.8 | 7.19 | 200 | 65135.1 | 0.004 | ng/L |
|  | linuron | herbicides |  | LC-MS | 3.2 | 3.19 | 2 | 651.4 | 0.422 | µg/L |
|  | malathion | insecticides |  | GC-MS | 2.36 | 2.26 | 17.5 | 5699.3 | 31.642 | ng/L |
|  | mandipropamid | fungicides |  | LC-MS | 3.2 | 3.19 | 0.6 | 195.4 | 0.126 | µg/L |
|  | MCPA | herbicides | dissociated | LC-MS | 3.25 | 3.24 | 5 | 1628.4 | 0.927 | µg/L |
|  | MCPB | herbicides | dissociated | LC-MS | 2.79 | 2.73 | 10 | 3256.8 | 6.018 | µg/L |
|  | MCPP-p | herbicides | dissociated | LC-MS | 3.2 | 3.19 | 3 | 977.0 | 0.632 | µg/L |
|  | mesosulfuron-methyl | herbicides | dissociated | LC-MS | -0.48 | -0.90 | 0.3 | 97.7 | 776.079 | µg/L |
|  | mesotrione | herbicides |  | LC-MS | 0.9 | 0.63 | 0.5 | 162.8 | 37.881 | µg/L |
|  | metalaxyl-m | fungicides |  | LC-MS | 1.75 | 1.58 | 0.6 | 195.4 | 5.166 | µg/L |
|  | metamitron | herbicides |  | LC-MS | 0.83 | 0.56 | 3 | 977.0 | 271.863 | µg/L |
|  | metazachlor | herbicides |  | LC-MS | 2.13 | 2.00 | 0.3 | 97.7 | 0.977 | µg/L |
|  | metconazole | fungicides |  | GC-MS | 3.85 | 3.91 | 750 | 244256.5 | 29.973 | ng/L |
|  | methabenzthiazuron | herbicides |  | LC-MS | 2.64 | 2.57 | 3 | 977.0 | 2.650 | µg/L |
|  | methiocarb | insecticides |  | LC-MS | 2.92 | 2.88 | 1 | 325.7 | 0.432 | µg/L |
|  | metobromuron | herbicides |  | LC-MS | 2.38 | 2.28 | 3 | 977.0 | 5.154 | µg/L |
|  | metolachlor | herbicides |  | LC-MS | 3.13 | 3.11 | 2 | 651.4 | 0.504 | µg/L |
|  | metoxuron | herbicides |  | LC-MS | 1.64 | 1.46 | 3 | 977.0 | 34.226 | µg/L |
|  | metrafenone | fungicides |  | GC-MS | 4.3 | 4.41 | 1.25 | 407.1 | 0.016 | µg/L |
|  | metribuzin | herbicides |  | LC-MS | 1.7 | 1.52 | 0.9 | 293.1 | 8.807 | µg/L |
|  | metsulfuron-methyl | herbicides |  | LC-MS | 2.2 | 2.08 | 0.2 | 65.1 | 0.545 | µg/L |
|  | mevinphos | insecticides |  | GC-MS | 0.13 | -0.22 | 50 | 16283.8 | 27162.959 | ng/L |
|  | monolinuron | herbicides |  | LC-MS | 2.3 | 2.19 | 2 | 651.4 | 4.216 | µg/L |
|  | Monuron | herbicides |  | LC-MS | 1.94 | 1.79 | 3 | 977.0 | 15.886 | µg/L |
|  | nicosulfuron | herbicides | dissociated | LC-MS | 0.35 | 0.02 | 0.4 | 130.3 | 123.772 | µg/L |
|  | Oxadiazon | herbicides |  | LC-MS | 5.33 | 5.56 | 10 | 3256.8 | 0.009 | µg/L |
|  | oxydemeton-methyl | insecticides |  | LC-MS | -0.74 | -1.19 | 1 | 325.7 | 5031.225 | µg/L |
|  | parathion-ethyl | insecticides |  | GC-MS | 3.83 | 3.89 | 75 | 24425.6 | 3.155 | ng/L |
|  | parathion-methyl | insecticides |  | GC-MS | 3 | 2.97 | 75 | 24425.6 | 26.374 | ng/L |
|  | pencycuron | fungicides |  | LC-MS | 4.68 | 4.83 | 0.7 | 228.0 | 0.003 | µg/L |
|  | pendimethalin | herbicides |  | GC-MS | 5.2 | 5.41 | 150 | 48851.3 | 0.190 | ng/L |
|  | pentachlorophenol | insecticides | dissociated | LC-MS | 5.12 | 5.32 | 20 | 6513.5 | 0.031 | µg/L |
|  | phenmedipham | herbicides |  | LC-MS | 3.59 | 3.62 | 4 | 1302.7 | 0.311 | µg/L |
|  | phtalimide | fungicides |  | GC-MS | 3.01 | 2.98 | 25 | 8141.9 | 8.569 | µg/L |
|  | pirimicarb | insecticides |  | LC-MS | 1.7 | 1.52 | 50 | 16283.8 | 489.252 | ng/L |
|  | pirimiphos-methyl | insecticides |  | LC-MS | 4.12 | 4.21 | 100 | 32567.5 | 2.003 | ng/L |
|  | prochloraz | fungicides |  | LC-MS | 3.5 | 3.52 | 2 | 651.4 | 0.196 | µg/L |
|  | propamocarb | fungicides | protonated | LC-MS | -1.3 | -1.81 | 4 | 1302.7 | 84324.924 | µg/L |
|  | propiconazool | fungicides |  | GC-MS | 3.72 | 3.77 | 150 | 48851.3 | 8.360 | ng/L |
|  | Propoxur | insecticides |  | LC-MS | 1.52 | 1.32 | 0.6 | 195.4 | 9.305 | µg/L |
|  | prosulfocarb | herbicides |  | GC-MS | 4.65 | 4.80 | 50 | 16283.8 | 0.258 | ng/L |
|  | prothioconazole | fungicides |  | LC-MS | 2 | 1.86 | 30 | 9770.3 | 136.254 | µg/L |
|  | pyraclostrobin | fungicides |  | LC-MS | 3.99 | 4.07 | 0.5 | 162.8 | 0.014 | µg/L |
|  | pyraflufen-ethyl | herbicides |  | GC-MS | 3.49 | 3.51 | 750 | 244256.5 | 75.290 | ng/L |
|  | Pyridaben | insecticides |  | GC-MS | 6.37 | 6.71 | 1 | 325.7 | 0.000 | µg/L |
|  | pyroxsulam | herbicides |  | LC-MS | -1.01 | -1.49 | 0.6 | 195.4 | 6023.168 | µg/L |
|  | quinoxyfen | fungicides |  | GC-MS | 5.1 | 5.30 | 75 | 24425.6 | 0.122 | ng/L |
|  | quizalofop-p-ethyl | herbicides |  | GC-MS | 4.61 | 4.76 | 500 | 162837.7 | 2.859 | ng/L |
|  | rimsulfuron | herbicides | dissociated | LC-MS | -1.46 | -1.99 | 0.6 | 195.4 | 19046.930 | µg/L |
|  | Simazine | herbicides |  | GC-MS | 2.18 | 2.06 | 200 | 65135.1 | 573.138 | ng/L |
|  | sulcotrione | herbicides |  | LC-MS | 2.31 | 2.20 | 0.7 | 228.0 | 1.438 | µg/L |
|  | tebuconazole | fungicides |  | GC-MS | 3.7 | 3.74 | 125 | 40709.4 | 7.332 | ng/L |
|  | tepraloxydim | herbicides |  | LC-MS | 1.5 | 1.30 | 2 | 651.4 | 32.645 | µg/L |
|  | terbuthylazine | herbicides |  | GC-MS | 3.4 | 3.41 | 500 | 162837.7 | 63.189 | ng/L |
|  | Terbutryn | herbicides |  | GC-MS | 3.74 | 3.79 | 75 | 24425.6 | 3.972 | ng/L |
|  | thiabendazole | fungicides |  | LC-MS | 2.39 | 2.29 | 6 | 1954.1 | 10.047 | µg/L |
|  | thiacloprid | insecticides |  | LC-MS | 1.26 | 1.03 | 0.6 | 195.4 | 18.097 | µg/L |
|  | thiamethoxam | insecticides |  | LC-MS | -0.13 | -0.51 | 0.3 | 97.7 | 316.969 | µg/L |
|  | tolclofos-methyl | fungicides |  | LC-MS | 3.8 | 3.86 | 8 | 2605.4 | 0.363 | µg/L |
|  | tolyfluanide | fungicides |  | GC-MS | 3.9 | 3.97 | 500 | 162837.7 | 17.583 | ng/L |
|  | triadimenol | fungicides |  | LC-MS | 3.18 | 3.17 | 3 | 977.0 | 0.666 | µg/L |
|  | tri-allate | herbicides |  | GC-MS | 4.6 | 4.74 | 50 | 16283.8 | 0.293 | ng/L |
|  | triazophos | insecticides |  | GC-MS | 3.55 | 3.58 | 150 | 48851.3 | 12.915 | ng/L |
|  | trifloxystrobin | fungicides |  | LC-MS | 4.5 | 4.63 | 0.5 | 162.8 | 0.004 | µg/L |
|  | Trifluralin | herbicides |  | GC-MS | 5.34 | 5.57 | 50 | 16283.8 | 0.044 | ng/L |
|  | triflusulfuron-methyl | herbicides | dissociated | LC-MS | 3.94 | 4.01 | 0.8 | 260.5 | 0.025 | µg/L |
|  | trinexapac-ethyl | herbicides |  | LC-MS | -0.29 | -0.69 | 0.8 | 260.5 | 1272.810 | µg/L |
|  | tritosulfuron | herbicides | dissociated | LC-MS | 2.93 | 2.89 | 0.6 | 195.4 | 0.252 | µg/L |

**Table S4.** Chromatographic details of organic compound analysis. Data obtained from de Baat et al., 2019.

| **Contaminant analysis**  (supplier of standard) | **Chromatographic separation** | **Detection** |
| --- | --- | --- |
| - Phenanthrene   (J.T. Baker)   - pyrene   (J.T. Baker) | - **System:** Prominence, Shimadzu, Kyoto, Japan - **Column:** LiChrospher C18 column (5µm; 250 x 4 mm; Knauer, Berlin, Germany). - **Injection:** 20 µL injected of acetonitrile diluted two times in ultrapure water. | - **System:** Prominence Fluorescence detecter, Shimadzu, Kyoto, Japan - **Quantification:** PAH concentrations were quantified with external standard calibrations. |
| - HHCB   (International Flavors & Fragrances, Hilversum, NL)   - nonylphenol   (Acros Organics, NJ, USA   - triclosan   (Sigma-Aldrich)   - methyl-triclosan   (Dr Ehrenstorfer GmbH, Augsburg, Germany) | - **System:** ThermoQuest Trace GC 2000 (Thermo Fisher Scientific) - **Column:** DB-5MS fused silica column (60 m × 0.25 mm, 0.25 μm film thickness; J&W Scientific, Folsom, CA, USA). - **Injection:** 1 µL of the extracts was directly injected cold on-column. | - **System:** Finnigan Trace MS quadrupole MS (Thermo Fisher Scientific) - **Quantification:** Selected ion monitoring (SIM), identification was based on retention time and three or four (nonylphenol) compound specific masses and external standard calibrations. |
| - BPA   (Sigma-Aldrich) | - **System:** Prominence, Shimadzu, Kyoto, Japan - **Column:** Kinetex 5 µm (2.1 x 50 mm) - **Injection:** 20 μL injected of acetonitrile diluted three times in ultrapure water | - **System:** QTRAP 4000 MS system (AB SCIEX, MA, USA) - **Mode:** negative electrospray ionization (ESI) - **Quantification:** BPA concentrations were quantified with external standard calibrations. |
| - Pesticides via LC-MS/MS | - **System:** Accela 1250 HPLC system (Thermo Fisher Scientific) equipped with a CTC autosampler - **Column:** Hypersil GOLD aQTM SPE column (20 X 2.1 mm, 12 µm) + Hypersil GOLDTM HPLC column (50 X 2.1 mm, 3 µm), both Thermo Fisher Scientific) **Injection:** 1 μL | - **System:** TSQ Vantage triple quadrupole mass spectrometer - **Mode:** negative and positive mode with an electrospray ionization source (Thermo Fisher Scientific). - **Quantification:** selected reaction monitoring (SRM), and multiple external standards |
| - Pesticides via GC-MS | - **System:** Trace GC system (Thermo Fisher Scientific) - **Column:** DB-5MS fused silica column (30 m X 0.25 mm, 0.,25 µm film thickness, J&W Scientific). | - **System:** Dual stage quadrupole (DSQ) MS (Thermo Fisher Scientific) - **Quantification:** selected ion recording (SIR) mode, and multiple external standards |

**Table S5.** Freely dissolved contaminant concentrations detected in sediments from sites with different land uses. n.m. = not measured. - = not detected. Data obtained from de Baat et al., 2019.

**SI 1.**Toxic unit calculations

As for *C. riparius*, no sufficiently homogeneous dataset with effect concentrations was available for reliable TU calculation, reported acute (48 h) 50% toxic effect concentrations (EC50) of the detected compounds to *Daphnia magna* were used, given the extensive data availability for this species and its common use as model organism for toxicity to aquatic invertebrate. Cumulative TUs were calculated by summing the lowest reported TU values for separate compounds per location assuming response additivity, in which TU was defined as the ratio of the measured concentration of a given compound to its EC50. Data obtained from de Baat et al., 2019).

**SI 2.** Culture set up test organisms.

Larvae of the caddisfly *Sericostoma personatum* were collected from woodland streams in the National Park the Hoge Veluwe (Table S3) by sieving the sediment (1 mm mesh size), after which they were transferred into the laboratory to set up a culture. The *S. personatum* culture consisted of a 20 L aquarium containing a 5 cm layer of sediment with twigs and leaves from the sampling site, supplemented with surface water from the sampling site to ease the transition from field to laboratory. The culture was fed twice a week with beech and oak leaves collected at a site close to Amsterdam (Table S3), which were incubated for at least 7 days in surface water from the woodland streams where *S. personatum* was collected. Juveniles of the isopod *Asellus aquaticus* originated from water bodies surrounding Amsterdam (Table S3) and were collected using a hand net, after which they were transferred into a 20 L aquarium, filled with surface water and leaves and twigs from the sampling site. The culture was fed twice a week with beech and oak leaves collected at a location close to Amsterdam (Table S3), which were incubated for at least 7 days in surface water from the ditches where the *A. aquaticus* juveniles were collected. Larvae of the non-biting midge *Chironomus riparius* originated from the University of Amsterdam in-house laboratory culture. The culture was maintained in several 20 L aquaria containing quartz sand overlaid with Dutch standard water (DSW) (Marinković et al., 2011). The culture was fed a mixture of Trouvit (Trouw, Fontaine-les-Vervins, France) and Tetraphyll (Tetrawerke, Melle, Germany) in a ratio of 20:1. This mixture was also used as food in the whole sediment bioassays. All cultures were kept at 20 ± 1 ˚C, 65% humidity, a 16:8 h light:dark photoperiod and the water was aerated constantly.

*Marinković, M., Verweij, R. A., Nummerdor, G. A., Jonker, M. J., Kraak, M. H. S., & Admiraal, W. (2011). Life cycle responses of the midge chironomus riparius to compounds with different modes of action. Environmental Science and Technology, 45(4), 1645–1651.*

**Table S6.** Overview of the sampling locations of test organisms and leaves.

| **test organism** | **Location** | **GPS coordinates** | **sampling day (dd/mm/yy)** |
| --- | --- | --- | --- |
| *Sericostoma personatum* | Nationaal Park de Hoge Veluwe | 51.974660, 5.815557 | 05/04/18 |
| *Asellus aquaticus* | Twiske | 52.439820, 4.894512 | 23/04/18 |
| Beech and oak leaves | Twiske | 52.439820, 4.894512 | 23/04/18 |
|  |  |  |  |

**Table S7.** Quality control measurements (average ± st.err), i.e. pH, O_2_, conductivity and NH_4_^+^on day 28 of the experiment. * 1 core omitted from analyses.

*
